# Determinants of Psychosocial and Mental Health Risks of Multicultural Adolescents: A Multicultural Adolescents Panel Study 2023

**DOI:** 10.3390/healthcare13192409

**Published:** 2025-09-24

**Authors:** Jeoungmi Kim, Vasuki Rajaguru

**Affiliations:** 1 Department of Nursing Science, Kaya University, Gimhae 50830, Republic of Korea; jeoung66@kaya.ac.kr; 2 Department of Healthcare Management, Graduate School of Public Health, Yonsei University, Seoul 03722, Republic of Korea

**Keywords:** multicultural adolescents, psychosocial and mental health risks, depression, self-esteem, aggression, Republic of Korea, MAPS

## Abstract

Background: Adolescence is a critical developmental period when Psychosocial and mental health risks such as depression, social withdrawal, low self-esteem, and aggression may shape lifelong mental health outcomes. In Republic of Korea, multicultural adolescents face additional vulnerabilities due to cultural identity struggles, discrimination, and family stressors. Objective: This study examined the determinants of Psychosocial and mental health risks among multicultural adolescents using data from the 2023 Multicultural Adolescents Panel Study (MAPS). Methods: A cross-sectional analysis was conducted with adolescents from multicultural families. Outcomes included social withdrawal, depression, self-esteem, and aggression, measured by four-point Likert scales. Covariates comprised sociodemographic factors such as sex, age, parental nationality, parental education, parental occupation, and household income. Partial correlations, F-tests, and multiple linear regression were used to identify significant predictors. Results: Female adolescents reported significantly lower self-esteem (*p* = 0.003). Region was associated with both self-esteem (*p* = 0.037) and aggression (*p* = 0.047), with adolescents living in metropolitan areas reporting lower self-esteem and higher aggression compared to those in capital areas. Non-Korean father nationality increased the likelihood of aggression (*p* = 0.036), while higher paternal education reduced aggression risk (*p* = 0.048). Overweight and obesity were linked to greater aggression (*p* = 0.007, *p* = 0.050, respectively). Conclusions: The findings highlight the interrelated nature of Psychosocial and mental health risks among multicultural adolescents and underscore the influence of gender, family background, and body image. Addressing these factors within culturally sensitive school and community interventions is essential to support positive Psychosocial and mental health risk outcomes.

## 1. Introduction

Adolescence is a critical stage for identity formation, peer relationships, and psychological well-being, yet mental health problems such as depression, anxiety, and social withdrawal remain leading causes of disability worldwide [1,2,3]. Psychosocial and mental health risk factors including low self-esteem, family conflict, and peer victimization are consistently linked to poor outcomes [4,5]. In Republic of Korea, the number of multicultural adolescents has more than tripled in the past decade, now accounting for nearly 3% of students [6]. These youth face unique challenges of cultural identity, discrimination [7], and language barriers in a largely homogeneous society, placing them at heightened risk for depression and social withdrawal. Prior studies demonstrate that multicultural adolescents in Republic of Korea are at increased risk of depression [8], social withdrawal, bullying, and low self-esteem compared to their peers from non-multicultural backgrounds [9,10]. Social exclusion, peer victimization, and linguistic or cultural differences contribute to difficulties in school adjustment and peer integration, which are strong predictors of Psychosocial and mental health outcomes [11,12]. Furthermore, parental factors such as maternal education, father’s employment stability, and family income strongly influence adolescent well-being, with socioeconomic inequalities exacerbating of Psychosocial and mental health risk outcomes [8,13,14].

The Multicultural Adolescents Panel Study (MAPS), conducted by the National Youth Policy Institute, provides nationally representative cross-sectional data for secondary analysis to examine these issues in Republic of Korea. Analyses of MAPS have shown that individual level factors such as stress, self-esteem, and life satisfaction, alongside social factors like peer and parental support, play pivotal roles in shaping mental health outcomes [6,15]. Moreover, experiences of discrimination and acculturative stress have emerged as significant predictors of depressive symptoms and social withdrawal [11,13]. Low self-esteem has been associated with a higher risk of depression and social withdrawal, while those with positive self-perceptions demonstrate resilience in the face of acculturative challenges [15].

International research reinforces these findings, showing that minority and migrant adolescents worldwide experience higher risks of depression and psychological distress due to acculturation stress, peer victimization, and systemic inequalities [16,17]. Social withdrawal, often arising from bullying or exclusion, is linked to loneliness, poor school adjustment, and future mental health disorders [12]. Similarly, global evidence underscores the centrality of self-esteem in adolescent adjustment, mediating the effects of stress and social adversity on psychological well-being [18].

Understanding the determinants of Psychosocial and mental health risks in multicultural adolescents is essential for designing effective interventions. Internal Psychosocial and mental health factors, particularly depression and aggression, reflect emotional and behavioral patterns that directly influence adolescent well-being. Depression, one of the most common and debilitating conditions in adolescence, is closely linked to discrimination, acculturative stress, and limited social support [16,17,19]. Aggression represents an externalizing response to frustration, stigma, and chronic stressors, often associated with peer victimization and academic pressures [1,20,21]. Both depression and aggression have long-term consequences for educational achievement, employment, and adult health outcomes if left unaddressed [2,4].

External social stressors including social withdrawal and low self-esteem shape how adolescents interact with their environment and how they are perceived by peers. Social withdrawal, often arising from bullying, exclusion, or perceived discrimination, can intensify isolation and vulnerability to mental health problems [6,13,15,22]. In contrast, self-esteem acts as a protective factor moderating the impact of stressors, influencing whether adolescents adapt successfully or become overwhelmed by challenges [3,12,23,24]. In Republic of Korea, where academic competition and conformity are emphasized, multicultural adolescents may experience heightened pressures that erode self-esteem and intensify Psychosocial and mental health risks. Overall, evidence indicates that depression, social withdrawal, and self-esteem are interrelated domains that critically shape the Psychosocial and mental health well-being of multicultural adolescents. Depression often emerges from experiences of discrimination and cultural stress, while social withdrawal reinforces isolation and self-esteem determines resilience or vulnerability to these pressures. In the Republic of Korean context, examining these dynamics through MAPS offers valuable insight into how individual and social factors interact across developmental stages. This study builds upon and replicates key findings from previous research on Psychosocial and mental health risks among multicultural adolescents, including the links between low self-esteem, withdrawal, and depression [6,16]. By using nationally representative MAPS data, it extends prior studies by testing these associations within the Republic of Korean context and across a broader range of sociodemographic factors.

Therefore, this study aims to identify sociodemographic and parents’ cultural determinants of mental health risks including social withdrawal, depression, self-esteem, and aggression among multicultural adolescents in Republic of Korea. The findings are expected to inform inclusive youth mental health strategies and targeted interventions at both community and policy levels.

## 2. Materials and Methods

### 2.1. Study Design and Data Source

This study was based on data from the 2023 wave of the Multicultural Adolescents Panel Study (MAPS), conducted by the National Youth Policy Institute (NYPI) in Republic of Korea. MAPS is a nationally representative, longitudinal panel survey that follows children and adolescents from multicultural families to track their Psychosocial and mental health, educational, and health-related outcomes, from 17 cities and provinces across Republic of Korea, ensuring broad geographic coverage. The survey uses structured questionnaires administered through trained interviewers and is designed to capture both individual and contextual factors influencing the well-being of multicultural youth.

The NYPI provided the study data after the research proposal was submitted. All personal identifiers were excluded to ensure confidentiality. This analysis did not require IRB approval from NYPI; however, all participants and their guardians gave informed consent at the time of data collection. Declaration of Helsinki ethical norms, including confidentiality and data protection, were followed in all procedure

### 2.2. Study Participants

**Figure 1:**
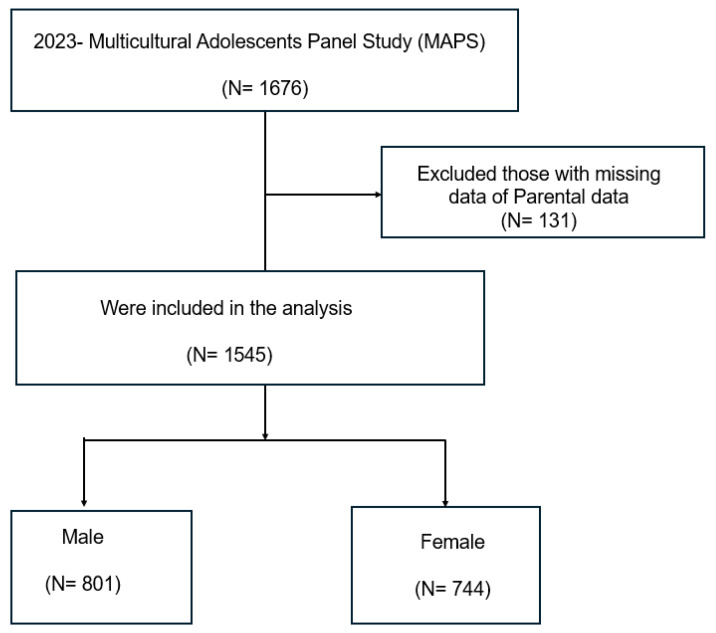
Flowchart for selection of study participants.

A total of 1676 adolescents aged 13–16 years participated in the 2023 survey wave. After excluding participants with incomplete responses or missing data on key study variables, 1545 respondents were retained for the final analysis. The inclusion criteria were (1) adolescents belonging to multicultural families, defined as having at least one parent of non-Korean origin, and (2) completion of the mental health risks on self-esteem, social withdrawal, depression, and aggression. Exclusion criteria included incomplete demographic information or non-response to outcome variables (Figure 1).

### 2.3. Measures

#### 2.3.1. Outcome Variables

Four Psychosocial and mental health risks outcomes were included. Self-esteem was measured on a four-point Likert type scale ranging from “Not at all” to “Very good,” with higher scores indicating more positive self-perceptions. Social withdrawal was assessed using a four-point scale from “Strongly disagree” to “Strongly agree,” reflecting the extent of social isolation and disengagement. Depression was evaluated on a four-point scale ranging from “Not at all” to “Very often,” with lower ratings indicating depressive symptoms. Aggression was measured through self-reported behavioral tendencies toward irritability, hostility, or confrontation, scored on a four-point Likert scale, with higher scores indicating greater aggression.

#### 2.3.2. Covariates

Sociodemographic and family-related characteristics were included as control variables. This included sex (male, female), age (13–16 years), and province of residence (urban, rural). Parental variables consisted of age (16–20 years), education (middle school or less, high school, undergraduate or above), occupation (professional/managerial, technical, others), and country of birth (Korean and non-Korean). Household economic status was represented by average monthly income (KRW ≤ 300, 301–600, 601–900, ≥901/10,000). Body mass index (BMI) was calculated using the standard formula: weight in kilograms divided by the square of height in meters (kg/m^2^). BMI was also categorized into four groups according to the conventional WHO classification [25] underweight (<18.5 kg/m^2^), normal weight (18.5–24.9 kg/m^2^), overweight (25–29.9 kg/m^2^), and obese (≥30 kg/m^2^). This allowed the analysis to consider physical health status as a potential predictor of Psychosocial and mental health risk outcomes.

### 2.4. Data Analysis

Data was collected and missing covariate data were excluded using listwise deletion, resulting in a final analytic sample of 1545 adolescents. All the final data were analyzed in four steps. First, descriptive statistics (frequencies, percentages, means, and standard deviations) were computed to summarize the demographic and socioeconomic characteristics of the participants, as well as the distribution of outcome variables (self-esteem, social withdrawal, depression, and aggression). Second, correlations were performed to assess the relationships among the Psychosocial and mental health risk variables and between the key covariates, including age, sex, parental education, income, and body mass index. Third, Pearson’s correlation coefficients were calculated for continuous variables, while chi square tests were used for categorical variables. Finally, multiple linear regression analyses were conducted to identify significant predictors of Psychosocial and mental health risk outcomes. All the data were analyzed using IBM SPSS Statistics (version 25). Statistical significance was set at *p* < 0.05, and standardized regression coefficients (β) were reported to indicate the strength and direction of associations.

## 3. Results

### 3.1. General Demographic Characteristics of Female Students

Table 1 summarizes the characteristics of 1545 adolescents from multicultural families. Slightly more than half were male (51.8%), and nearly all were aged 14 (94.5%). Most lived in metropolitan areas (53.1%). Mothers were mainly in their 40s (47.6%) and fathers in their 50s or older (97.3%). Parental education was concentrated at the high school level (mothers 44.7%, fathers 60.5%), with mothers largely in “other” jobs (54.1%) and fathers distributed across professional (20.7%), technical (33.1%), and other (46.2%) roles. The majority of households earned KRW 301,000–600,000 monthly (70.6%). Comparing the sample’s household income distribution to the national median indicated that most households fell within the lower-middle income range. Most adolescents had normal BMI (59.0%), with 25.4% underweight. Mean mental health scores were 1.83 (aggression), 2.18 (social withdrawal), 1.56 (depression), and 3.21 (self-esteem).

### 3.2. Relationship Between Psychosocial and Mental Health Risk and General Characteristics of Multicultural Adolescents

Table 2 shows that among multicultural adolescents, regions had a highly significant effect on social withdrawal (F = 15.956, *p* < 0.001), with those in other regions reporting higher social withdrawal scores (M = 2.352, SD = 0.808) compared to those in capital/metropolitan areas (M = 2.064, SD = 0.776). Mother’s education also significantly influenced social withdrawal (F = 5.017, *p* = 0.007), with adolescents of mothers having middle school or less education reporting the highest scores (M = 2.275, SD = 0.798). For depression, sex was significant (t = 5.991, *p* = 0.014), with females (M = 1.596, SD = 0.618) scoring higher than males (M = 1.524, SD = 0.540). Parental occupation also affected depression (t = 3.104, *p* = 0.002), with nonprofessional mothers’ children having more depression. Fathers’ education was significant (F = 3.080, *p* = 0.046), as adolescents with less educated fathers had higher depression scores.

### 3.3. Correlation Between Psychosocial and Mental Health Risks of Multiculture Adolescents

Table 3 presents the correlations among mental health risk variables in adolescents from multicultural families. All three negative Psychosocial outcomes—aggression, social withdrawal, and depression—are significantly and positively related to one another, with the strongest association observed between depression and social withdrawal (r = 0.537, *p* < 0.01) and between depression and aggression (r = 0.492, *p* < 0.01). This indicates that adolescents who report higher levels of one problem behavior are more likely to exhibit others simultaneously. In contrast, self-esteem is significantly and negatively correlated with all three risk factors—aggression (r = −0.119, *p* < 0.01), social withdrawal (r = −0.211, *p* < 0.01), and depression (r = −0.373, *p* < 0.01).

### 3.4. Factors Affecting the Psychosocial and Mental Health Risks of Multiculture Adolescents

Table 4 shows the regression results for factors influencing Psychosocial and mental health risks among multicultural adolescents. The intercept for social withdrawal was 1.58 (SE = 0.85, 95%CI = 0.00–3.25; t = 1.86, *p* = 0.049) and 1.11 for depression (SE = 0.64, 95% CI = −0.14–2.36; t = 1.74, *p* = 0.081). Female adolescents had slightly higher depression scores than males (B = 0.076, SE = 0.030, 95% CI = 0.016–0.135; t = 2.51, *p* = 0.012) but showed no significant difference in social withdrawal. Age effects were generally nonsignificant. Living in metropolitan or other regions was associated with significantly higher social withdrawal compared with the capital (B = 0.141 and 0.287; *p* = 0.002 and <0.001, respectively), while regional effects on depression were nonsignificant. Parental nationality, most parental age groups, and parental occupation were not associated with either outcome. Higher maternal education (high school vs. middle school) was related to greater social withdrawal (B = 0.142, *p* = 0.004), whereas paternal undergraduate education was marginally associated with higher depression (B = 0.108, *p* = 0.049). BMI showed a small positive association with depression (B = 0.075, SE = 0.030; 95% CI = 0.016–0.134; t = 2.51; *p* = 0.012) but not with social withdrawal.

Table 5 shows the multiple logistic regression analysis of factors influencing self-esteem and aggression among multicultural adolescents. Self-esteem was 3.36 (SE = 0.62, 95% CI = 2.14–4.59; t = 5.39, *p* < 0.001) and aggression was 1.76 (SE = 0.69, 95% CI = 0.41–3.10; t = 2.55, *p* = 0.011). Female adolescents had significantly lower self-esteem than males (B = −0.089, 95% CI = −0.147 to −0.031; *p* = 0.003) but showed no difference in aggression. Age effects were small and nonsignificant for both outcomes. Living in metropolitan areas was linked to lower self-esteem (B = −0.070, *p* = 0.037) but higher aggression (B = 0.073, *p* = 0.047), whereas living in “other” regions was not significant. Non-Korean maternal nationality was not associated with self-esteem but showed a small positive effect on aggression (B = 0.021, 95% CI = 0.001–0.043; *p* = 0.004), and non-Korean paternal nationality was marginally related to higher aggression (B = 0.094, *p* = 0.036). Most parental age groups and education levels showed no clear effects, although paternal undergraduate education was negatively related to aggression (B = −0.117, *p* = 0.048). Mother’s and father’s occupation categories were generally nonsignificant, except that fathers classified as “others” showed slightly higher aggression scores (B = 0.079, 95% CI = 0.009–0.168; *p* = 0.017). Monthly household income did not predict either outcome. For BMI, overweight status was associated with higher aggression (B = 0.152, 95% CI = 0.042–0.263; *p* = 0.007), and obesity also predicted greater aggression (B = 0.185, 95% CI = 0.000–0.371; *p* = 0.005), but BMI categories were not related to self-esteem.

## 4. Discussion

This study explored Psychosocial and mental health risks in multicultural adolescents in Korea, highlighting how individual, familial, and sociocultural factors shape self-esteem, aggression, social withdrawal, and depression. The results showed that both individual (sex, age, and BMI) and familial (parental nationality, education, and occupation) factors significantly shaped these outcomes.

The findings indicate that female adolescents exhibited lower self-esteem and higher levels of depression compared to males. However, the multiple linear association regression revealed contrasting patterns across different Psychosocial and mental health risk factors. This finding aligns with global evidence of greater vulnerability to internalizing disorders among adolescent girls [18,21]. In Korea, these differences may be intensified by strong sociocultural expectations related to body image, academic achievement, and gender roles. Korean adolescents, particularly girls, are frequently exposed to rigid beauty standards and intense pressure from peers and media, contributing to body dissatisfaction and depressive symptoms [26], which provided evidence for sociocultural expectations and academic pressures contributing to lower self-esteem and higher depression among adolescent girls [10,26]. While some cross-national studies suggest narrowing gender gaps in adolescent depression [5], the persistence of high academic and appearance pressures in Korea may explain the heightened risk among girls.

The relationship among Psychosocial and mental health risk demonstrated significant associations between self-esteem, social withdrawal, aggression, and depression. These results reflect prior findings that low self-esteem is a key vulnerability factor for both internalizing and externalizing problems [3,27]. In Korea, self-esteem is closely tied to academic success due to the highly competitive college entrance system [15]. Adolescents who underperform academically often experience diminished self-worth, which increases their susceptibility to depression and social withdrawal [12,28]. This context may explain why self-esteem plays such a pivotal role in predicting Psychosocial and mental health risks in Korean adolescents.

Moreover, factors affecting the mental health risk outcomes showed that regional differences in adolescents in metropolitan and capital areas experienced greater social withdrawal compared with their rural counterparts. Urban youth in Korea are exposed to higher academic competition, longer study hours, and stronger peer comparison [20,22], all of which elevate stress and emotional difficulties. Conversely, adolescents in non-metropolitan regions may benefit from stronger community cohesion and less competitive schooling environments. However, international evidence shows that rural adolescents in some contexts suffer more due to limited mental health resources [19], suggesting that the Korean pattern may be context specific.

Parental factors emerged as particularly influential. Adolescents with non-Korean fathers reported lower self-esteem and higher aggression, highlighting challenges in bicultural identity formation and possible experiences of discrimination [3,15,16,23,24,29]. Parental nationality emerged as another critical predictor. Adolescents with non-Korean fathers reported lower self-esteem and higher aggression, echoing findings that multicultural adolescents in Korea face greater peer discrimination, identity conflict, and reduced social acceptance [15,29]. This is consistent with Korea’s relatively homogenous cultural history, where children of mixed ethnic backgrounds may encounter stigma. While multicultural education policies have been introduced, structural challenges persist, highlighting the need for culturally inclusive support systems.

Parental education and job type also significantly influenced outcomes. Higher parental education was linked to lower Psychosocial and mental health risks, supporting research that social and cultural capital provide resilience [27,30]. Yet, in Korea, this protective effect may coexist with risks, as highly educated parents often place extreme emphasis on academic performance, which can heighten adolescent stress and depression [26]. This dual effect suggests that interventions should balance educational support with stress management.

BMI was another significant factor, with overweight and obese adolescents demonstrating higher depression and aggression. Prior meta-analyses confirm that overweight youth experience greater bullying and emotional problems [10,28]. In Korea, these effects may be magnified due to cultural emphasis on thinness and widespread weight-based stigma [10]. Interestingly, some Western studies report more nuanced or even weaker associations between BMI and aggression, suggesting that cultural norms surrounding weight and appearance can moderate these effects [1,14]. In East Asian contexts such as Korea, Japan, and China, where collectivist norms and high parental expectations prevail, weight-related stigma tends to be stronger and more directly tied to peer relationships and school adjustment [7]. These cultural dynamics may explain why BMI emerged as a significant predictor of aggression in our sample. Future research should explore the mediating roles of peer victimization, self-esteem, and perceived discrimination to better understand how weight status translates into Psychosocial and mental health risks among multicultural adolescents.

Overall, these findings highlight that Psychosocial and mental health risks in multicultural adolescents are shaped by a complex interplay of gender, cultural identity, family background, and physical health. The results support prior evidence that multicultural youth are especially vulnerable due to both individual and structural factors, while also adding nuanced insights into the Korean context where paternal factors and body image pressures appear especially salient. In addition, parental factors were also influential: non-Korean father nationality increased aggression risk, higher paternal education was associated with reduced aggression, and maternal education patterns diverged from previous studies. Overweight and obesity were linked to greater aggression, highlighting the role of weight-related stigma. Depression, withdrawal, self-esteem, and aggression were moderately intercorrelated, underscoring shared vulnerabilities. Together, these findings show how gender, region, parental background, and BMI intersect to shape Psychosocial and mental health outcomes in multicultural adolescents. Thus, policy interventions in Korea should incorporate culturally sensitive family engagement, school-based stigma reduction, and mental health support tailored to the unique pressures faced by multicultural youth. Korean context, higher parental education among multicultural families may be linked to more stable socioeconomic status but also higher academic pressure on children, which could influence Psychosocial and mental health risks.

Future research should adopt longitudinal designs to better capture the causal pathways between sociodemographic factors and Psychosocial and mental health risks among multicultural adolescents. Expanding samples across diverse regions of Korea and including larger, nationally representative cohorts would enhance generalizability. Incorporating qualitative approaches, such as in-depth interviews or focus groups, could provide richer insights into cultural identity, acculturation challenges, and coping strategies.

This study has several limitations. First, its cross-sectional design restricts causal inference between sociodemographic factors and Psychosocial and mental health outcomes, making it difficult to establish directional relationships. Second, the use of self-reported measures may be influenced by biasing social desirability or underreporting, particularly in sensitive domains such as aggression and depression. Third, the sample was limited to adolescents in specific regions of Korea, which may reduce generalizability to all multicultural youth nationwide. Fourth, while multiple sociodemographic variables were considered, other potential confounding factors such as peer relationships, school environment, and community support were not assessed. Finally, the reliance on a 4-point Likert scale may have constrained the variability of responses, potentially underestimating nuanced differences in Psychosocial and mental health risks.

## 5. Conclusions

This study found that Psychosocial and mental health risks among multicultural adolescents in Korea, including depression, aggression, social withdrawal, and low self-esteem, are significantly influenced by gender, region, parental nationality and education, and BMI. Female adolescents, those from non-Korean parental backgrounds, and those with lower parental education reported greater vulnerabilities, while self-esteem served as a protective factor. These findings suggest that beyond universal adolescent challenges, Korea’s unique social pressures such as academic competitiveness, cultural homogeneity, and stigma toward multicultural identity exacerbate mental health risks. Therefore, culturally sensitive school and community-based interventions such as multiculture supportive services, public campaigns, etc., along with family and policy level support, are essential to promote resilience and ensure healthier development for multicultural youth in Korean society. Future research should adopt longitudinal designs to clarify the causal pathways between sociodemographic factors and Psychosocial and mental health risks among multicultural adolescents. Expanding the scope beyond the current MAPS cohort to include adolescents from diverse regions and family structures would improve generalizability. In addition, qualitative methods such as focus group interviews could enrich the understanding of cultural identity development, acculturation stress, and resilience mechanisms. In addition, further studies should also examine mediators such as peer victimization, parental support, and school integration to understand how these factors moderate the effects of gender, BMI, and parental background on Psychosocial and mental health outcomes.

## Figures and Tables

**Table 1:** General characteristics of multicultural adolescents.

Characteristics		Frequency	Percentage
Sex	Male	801	51.84
	Female	744	48.16
Age	13	15	0.97
	14	1460	94.5
	15	64	4.14
	16	6	0.39
Region	Capital and metropolitan city	471	30.5
	metropolitan areas	821	53.1
	Other	253	16.4
Mother nationality	1.0	524	33.9
	2.0	338	21.9
Father nationality	1.0	1305	84.5
	2.0	144	9.3
Mother age	30 s	639	41.36
	40 s	735	47.57
	≥50 s	171	11.07
Father age	30 s	30	1.94
	40 s	12	0.78
	≥50 s	1503	97.28
Mother education	Middle school or less	471	30.49
	High school	690	44.66
	Undergraduate and over	384	24.85
Father education	Middle school or less	216	13.98
	High school	934	60.45
	Undergraduate and over	395	25.57
Mother job	Professional or experts	223	14.43
	Technician or labor	487	31.52
	Others *	835	54.05
Father job	Professional or experts	319	20.65
	Technician or labor	512	33.14
	Others	714	46.21
Monthly Income (KRW 10,000)	<300	345	22.07
	301–600	1091	70.61
	601–900	99	6.41
	>901	14	0.91
BMI	Underweight	392	25.37
	Normal	911	58.96
	Overweight	190	12.3
	Obesity	52	3.37
Mental health problems (M ± SD)	Aggression	1.825	0.629
	Social withdrawal	2.183	0.778
	Depression	1.559	0.580
	Self-esteem	3.211	0.570

* Others, Chinese [Korean Chinese], Vietnam, Philippines, Japan, Thailand, other; M, Mean; SD, Standard deviation.

**Table 2:** Association between mental health risk in adolescents of multicultural families.

Variables	Social Withdrawal				Depression			
	M	SD	F/t	*p*	M	SD	F/t	*p*
Sex								
Male	2.171	0.750	0.405	0.525	1.524	0.540	5.991	0.014
Female	2.196	0.807			1.596	0.618		
AGE								
13	2.067	0.768	1.011	0.387	1.440	0.455	0.915	0.433
14	2.189	0.780			1.564	0.584		
15	2.115	0.733			1.494	0.504		
16	1.722	0.491			1.300	0.452		
Region								
Capital and metropolitan	2.064	0.776	15.956	0.000	1.528	0.561	1.888	0.170
Metropolitan areas	2.235	0.773			1.572	0.588		
other	2.352	0.808			1.577	0.573		
Mother nationality								
Korean	2.264	0.874	0.262	0.608	1.458	0.548	0.732	0.392
Non-Korean	2.182	0.776			1.560	0.580		
Father nationality								
Korea	2.182	0.777	0.009	0.925	1.571	0.591	3.540	0.060
China	2.187	0.781			1.497	0.519		
Mother age								
30 s	2.172	0.801	0.120	0.887	1.555	0.584	0.229	
40 s	2.191	0.762			1.556	0.573		
≥50 s	2.191	0.761			1.587	0.595		
Father age								
30 s	2.089	0.694	0.370	0.691	1.687	0.698	2.127	
40 s	2.306	0.881			1.833	0.894		
≥50 s	2.184	0.779			1.554	0.574		
Mother education								
Middle school or less	2.275	0.798	5.017	0.007	1.604	0.592	2.188	0.112
High school	2.129	0.748			1.532	0.560		
Undergraduate and over	2.169	0.797			1.552	0.598		
Father education								
Middle school or less	2.204	0.769	0.619	0.538	1.634	0.600	3.080	0.046
High school	2.194	0.775			1.561	0.581		
Undergraduate and over	2.146	0.788			1.513	0.563		
Mother job								
Professional or experts	2.172	0.801	0.325	0.722	1.552	0.615	0.104	0.002
Technician or labor	2.207	0.760			1.552	0.561		
Others	2.172	0.782			1.565	0.581		
Father job								
Professional or experts	2.186	0.777	0.056	0.945	1.542	0.572	0.310	0.734
Technician or labor	2.174	0.780			1.573	0.582		
Others	2.189	0.777			1.556	0.582		
Monthly Income								
<300	2.230	0.807	5.017	0.007	1.610	0.584	2.188	0.112
301–600	2.162	0.777			1.547	0.582		
601–900	2.229	0.709			1.517	0.535		
>901	2.405	0.573			1.557	0.652		
BMI								
Underweight	2.200	0.766	0.600	0.615	1.591	0.593	0.865	0.459
Normal	2.169	0.781			1.541	0.565		
Overweight	2.181	0.764			1.565	0.574		
Obesity	2.308	0.863			1.612	0.742		

M, Mean; SD, Standard Deviation.

**Table 3:** Correlation between psychosocial risks.

	Aggression	Social Withdrawal	Depression	Self-Esteem
Aggression	1	0.382 **	0.492 **	−0.119 **
Social withdrawal	0.382 **	1	0.537 **	−0.211 **
Depression	0.492 **	0.537 **	1	−0.373 **
Self-esteem	−0.119 **	−0.211 **	−0.373 **	1

** *p* < 0.01.

**Table 4:** Factors affecting the Psychosocial and mental health risks of social withdrawal and depression in multicultural adolescents.

Variables			Social Withdrawal						Depression			
	Estimate	SE	95%CI		t	*p*	Estimate	SE	95%CI		t	*p*
Intercept	1.583	0.850	0.002	0.332	1.862	0.049	1.10896	0.6358	−0.1382	2.35614	1.7441	0.081
Sex												
Male	1.000						1.000					
Female	0.019	0.040	−0.0603	0.0982	0.469	0.639	0.076	0.030	0.016	0.135	2.509	0.012
Age												
13	1.000						1.000					
14	0.124	0.202	−0.272	0.5214	0.615	0.539	0.124	0.151	−0.172	0.421	0.821	0.028
15	0.084	0.224	−0.355	0.5237	0.375	0.008	0.053	0.168	−0.276	0.381	0.315	0.753
16	0.379	0.379	−1.1221	0.363	0.251	0.317	0.469	0.283	0.324	0.786	0.598	0.050
Region												
Capital	1.000						1.000					
Metropolitan	0.141	0.045	0.052	0.229	3.110	0.002	0.041	0.034	−0.025	0.107	1.211	0.226
Others	0.287	0.062	0.164	0.409	4.608	<0.001	0.040	0.047	−0.051	0.131	0.851	0.395
Mother nationality												
Korean	1.000						1.000					
Non-Korean	0.067	0.167	−0.3948	0.261	0.399	0.690	0.069	0.125	−0.175	0.314	0.555	0.579
Father nationality												
Korean	1.000						1.000					
Non-Korean	0.011	0.055	−0.1191	0.097	0.192	0.847	0.069	0.041	−0.149	0.012	1.660	0.097
Mother age												
30 s	1.000						1.000					
40 s	0.071	0.045	−0.0169	0.158	1.582	0.114	0.033	0.033	−0.032	0.098	0.989	0.323
≥50 s	0.079	0.070	0.0572	0.082	1.140	0.048	0.063	0.052	−0.039	0.165	1.213	0.225
Father age												
30 s	1.000						1.000					
40 s	0.241	0.266	−0.2802	0.7629	0.908	0.364	0.172	0.199	−0.217	0.562	0.867	0.386
≥50 s	0.039	0.146	0.02469	0.0425	0.269	0.018	0.158	0.109	−0.371	0.056	1.444	0.149
Mother education												
Middle school or less	1.000						1.000					
High school	0.142	0.049	−0.238	−0.046	2.912	0.004	0.062	0.037	−0.133	0.0095	1.701	0.089
Undergraduate and over	0.100	0.062	−0.221	0.022	1.596	0.111	0.023	0.047	−0.115	0.0679	0.504	0.615
Father education												
Middle school or less	1.000						1.000					
High school	0.037	0.061	−0.082	0.157	0.607	0.544	0.057	0.046	−0.033	0.147	1.238	0.216
Undergraduate and over	0.011	0.073	−0.153	0.132	0.144	0.885	0.108	0.055	0.001	0.216	1.974	0.049
Mother job												
Professional or experts	1.000						1.000					
Technician or labor	0.015	0.068	−0.1179	0.147	0.217	0.828	0.024	0.051	−0.123	0.074	0.483	0.629
Others	0.007	0.062	−0.1294	0.1145	0.119	0.905	0.002	0.047	−0.089	0.092	0.032	0.974
Father job												
Professional or experts	1.000						1.000					
Technician or labor	0.064	0.059	−0.180	0.051	1.088	0.277	0.004	0.044	−0.082	0.090	0.095	0.924
Others	0.026	0.056	−0.135	0.083	0.462	0.644	0.017	0.008	0.001	0.034	0.411	0.011
Monthly Income												
<300	1.000						1.000					
301–600	0.495	0.787	−1.047	2.038	0.630	0.529	0.468	0.588	−0.631	1.676	0.796	0.046
601–900	0.438	0.786	−1.104	1.98	0.557	0.578	0.450	0.591	−0.685	1.621	0.761	0.447
>901	0.526	0.791	−1.024	2.076	0.665	0.506	0.517	0.608	−0.709	1.609	0.850	0.395
BMI												
Underweight	1.000						1.000					
Normal	0.034	0.047	−0.126	0.057	0.731	0.465	0.050	0.035	−0.119	0.019	1.420	0.156
Overweight	0.044	0.069	−0.179	0.092	0.628	0.530	0.024	0.052	−0.125	0.077	0.461	0.645
Obesity	0.088	0.117	−0.141	0.316	0.750	0.454	0.027	0.087	−0.144	0.197	0.304	0.761

**Table 5:** Factors affecting the Psychosocial and mental health risks of self-esteem and aggression in multicultural adolescents.

Predictor			Self-Esteem						Aggression			
	Estimate	SE	95%CI		t	*p*	Estimate	SE	95%CI		t	*p*
Intercept	3.363	0.623	2.1404	4.58695	5.393	<0.001	1.755	0.687	0.4064	3.10408	2.552	0.011
Sex												
Male	1.000						1.000					
Female	−0.0893	0.0296	−0.1474	−0.03118	−3.0137	0.003	−0.011	0.033	−0.075	0.053	−0.331	0.741
Age												
13	1.000						1.000					
14	−0.151	0.148	−0.442	0.14	−1.018	0.309	0.054	0.164	−0.267	0.375	0.329	0.743
15	−0.152	0.164	−0.475	0.169	−0.928	0.353	0.129	0.181	−0.227	0.484	0.710	0.478
16	0.389	0.277	−0.155	0.934	1.403	0.161	−0.092	0.306	−0.693	0.509	−0.300	0.764
Region												
Capital	1.000						1.000					
Metropolitan	−0.069	0.033	−0.134	−0.004	−2.093	0.037	0.073	0.037	0.001	0.145	1.986	0.047
Others	−0.036	0.045	0.125	0.053	−0.789	0.043	−0.038	0.050	−0.137	0.060	−0.763	0.445
Mother nationality												
Korean	1.000						1.000					
Non-Korean	0.071	0.122	−0.1696	0.31156	0.578	0.563	0.021	0.011	0.001	0.043	1.964	0.004
Father nationality												
Korean	1.000						1.000					
Non-Korean	0.055	0.04	−0.0241	0.13509	1.368	0.031	0.094	0.044	0.008	0.180	2.1136	0.036
Mother age												
30 s	1.000						1.000					
40 s	0.056	0.0327	0.018	0.089	1.737	0.083	0.042	0.036	−0.028	0.113	1.173	0.241
≥50 s	0.075	0.0511	0.045	0.098	1.468	0.142	0.002	0.056	−0.109	0.112	0.033	0.973
Father age												
30 s	1.000						1.000					
40 s	0.276	0.195	−0.1057	0.65933	1.419	0.156	0.308	0.215	−0.114	0.730	1.433	0.152
≥50 s	0.256	0.107	0.046	0.46587	2.391	0.017	0.174	0.118	−0.057	0.406	1.482	0.138
Mother education												
Middle school or less	1.000						1.000					
High school	0.019	0.035	−0.0507	0.09	0.548	0.584	0.003	0.039	−0.081	0.074	0.09	0.928
Undergraduate and over	0.021	0.045	−0.0686	0.11081	0.461	0.645	0.041	0.05	−0.140	0.058	0.815	0.415
Father education												
Middle school or less	1.000						1.000					
High school	−0.0148	0.044	−0.1028	0.07314	0.33	0.741	−0.071	0.049	−0.168	0.026	−1.427	0.154
Undergraduate and over	5.671	0.0535	−0.1044	0.1055	0.01	0.992	−0.117	0.059	−0.232	−8.06 × 10^−4^	−1.975	0.048
Mother job												
Professional or experts	1.000						1.000					
Technician or labor	0.028	0.049	−0.0685	0.12602	0.58	0.562	0.096	0.055	−0.011	0.204	1.764	0.078
Others	−0.0232	0.045	−0.1127	0.06625	0.509	0.611	0.050	0.050	−0.049	0.149	0.995	0.32
Father job												
Professional or experts	1.000						1.000					
Technician or labor	−0.0497	0.0433	−0.1347	0.03524	1.148	0.251	−0.050	0.048	−0.144	0.044	−1.047	0.295
Others	−0.0397	0.041	−0.1202	0.04074	0.968	0.333	0.079	0.045	0.009	0.168	1.767	0.017
Monthly Income												
<300	1.000						1.000					
301–600	−0.251	0.577	−1.383	0.880	0.435	0.663	−0.071	0.636	−1.318	1.177	−0.111	0.912
601–900	−0.202	0.577	−1.334	0.929	0.35	0.726	−0.131	0.636	−1.379	1.116	−0.207	0.836
>901	−0.106	0.580	−1.244	1.031	0.183	0.855	−0.098	0.639	−1.352	1.156	−0.153	0.879
BMI												
Underweight	1.000						1.000					
Normal	0.029	0.035	−0.039	0.097	0.833	0.405	0.001	0.038	−0.074	0.076	0.028	0.977
Overweight	0.082	0.051	−0.018	0.181	1.602	0.109	0.152	0.056	0.042	0.263	2.716	0.007
Obesity	0.066	0.086	0.077	0.077	0.331	0.041	0.185	0.094	0.000	0.371	1.964	0.005

## Data Availability

Data available in a publicly accessible repository. The data presented in this study are available upon request. Additional information about the MAPS dataset can be accessed at https://www.mrtc.re.kr/db/01.php (accessed on 22 August 2022).
